# Shoulder injuries in rugby players

**DOI:** 10.4103/0973-6042.50874

**Published:** 2009

**Authors:** Joe de Beer, Deepak N. Bhatia

**Affiliations:** Cape Shoulder Institute, Cape Town, South Africa

There has been an increase in the frequency and severity of shoulder injuries in rugby players in the recent years. This may be because the game has become more aggressive and intense and, over the years, the game has changed from being largely an amateur sport to one that is played at a professional level. Of equal importance is the fact that younger players are playing more competitively and aggressively, and there are specific injury patterns in these players with immature skeletal structures.

Why should one make separate mention of rugby injuries and not simply speak of shoulder injuries in general? The reasons are that certain injury patterns are specific to the sport. Rugby players have different requirements as far as rehabilitation is concerned as they need to return to this high contact activity within a relatively short period.

## STRUCTURES MOST OFTEN INJURED

### Soft tissue around the shoulder

In the game of rugby, direct blows are common and soft tissue bruising of the trapezius, the deltoid, and the pectoralis major muscles, and other soft tissue around the shoulder, occur regularly; however, this is usually not of long-term significance for the player.

### Acromioclavicular joint

Injuries to the Acromioclavicular (AC) joint are common and could be one of the most frequently occurring shoulder injuries in rugby. It usually results from a fall directly onto the shoulder, usually with the posterosuperior aspect of the shoulder striking the ground. This may result in either a sprain or dislocation of this joint due to rupture of the ligaments stabilizing the joint (i.e., the conoid, trapezoid, and AC ligaments). Grade I and grade II injuries are very common and are usually managed by team physicians and physiotherapists. Grade III injuries are also frequently seen and are generally referred to a specialist for an opinion. Grade IV and V injuries are less frequent in rugby players.

### Rotator cuff

The rotator cuff tendon is injured when the abducted or extended arm is forced downwards or backwards especially during a fall or a tackle. The injury may be a simple sprain or a frank tear of the tendon. The latter is relatively rare in the young player.

### Sternoclavicular joint

The Sternoclavicular (SC) joint is injured when the player falls directly onto the shoulder. The joint may be painfully swollen or frankly dislocated. Anterior subluxation of the joint is by far the most frequent; the more dangerous posterior subluxation is relatively rare.

### Glenohumeral joint

The shoulder joint itself may be injured during a direct fall onto the shoulder or when the horizontally abducted arm is forced posteriorly ('straight-arm tackle'). After an analysis of patients' descriptions of their falls and close examination of video clips of rugby games, we have come to the conclusion that this specific mechanism may be one of the causes for the high frequency of bony lesions in shoulder instability of rugby players. Another frequent cause of anterior dislocation is when a player falls forward with the elbow flexed, and the elbow contacts the ground first; as the body falls forwards, the arm is forced posteriorly and this results in an anterior dislocation. Anterior dislocations with bone loss, resulting in engaging Hill-Sachs lesions with anteroinferior loss of the glenoid bone ('inverted-pair glenoid'), are often seen in rugby. Simple Bankart lesions do not seem to be a frequent occurrence in rugby. HAGL (humeral avulsion of the glenohumeral ligaments) lesions are not uncommon in rugby. The mechanism is not entirely clear, but some players report that with the arm in forward elevation, an inferiorly directed force caused the onset of the pain and disability. It seems that in these players the mechanism causing the tearing of the inferior glenohumeral ligament may be an inferiorly directed force rather than the mechanism of abduction and external rotation. GLAD (glenolabral articular disruption) lesions occur commonly in rugby. The mechanism may be a direct fall onto the shoulder, causing disruption of the anterior surface of the glenoid articular cartilage and the labrum. Posterior dislocations of the humeral head do occur in rugby and are also associated with labral tears and bony lesions of the posterior rim of the glenoid. 'Reverse Hill-Sachs lesions' also occur but do not seem to be as relevant as the anterior engaging Hill-Sachs lesion. Another injury which could occur inside the joint is a bruise of the joint surface ('bone bruise') which may be sustained during a direct blow on the shoulder. This injury is apparent on MRI scan.

### Superior labrum anterior-to-posterior lesions

Superior labrum anterior-to-posterior (SLAP) lesions may be caused by a fall onto the elbow, resulting in an upward force into the shoulder joint. These lesions are not common in rugby and type II lesions are seen only on occasion. Type IV and V SLAP lesions, with the long head of the biceps tendon being involved or with a bucket-handle tear of the superior labrum, seem to be more common than type II lesions.

### Biceps tendon

The long head of the biceps muscle may be injured by traction forces on the flexed elbow. Rupture of the long head of the biceps is relatively rare; disinsertion of the distal tendon of the biceps at the elbow appears to be more common.

### Fractures

Fractures around the shoulder joint are relatively rare in adult players but they do occur regularly in young schoolboy players. Fractures of the clavicle and epiphyseal injuries of the humeral neck are common.

### Brachial plexus traction injuries

These usually occur when the head is forced away from the shoulder and the shoulder is pushed downwards, resulting in a severe stretch of the tissues between the shoulder and the neck. This may happen when a player falls forward and downwards, and the shoulder and the head make contact with the ground at the same time. This leads to stretching of the brachial plexus. This injury is referred to as a 'stinger' or 'burner' because the victim experiences a sudden severe burning pain down the arm.

### Pectoralis major tears

The tendon of the powerful pectoralis major muscle is very rarely torn. In rugby it could occur, for example, when a forward player has his arm engaging another player in a scrum, with the upper arm in abduction and the forearm around the adjacent player. When the scrum collapses, the contracting muscle tears off the tendon at its insertion.

## MANAGEMENT OF SHOULDER INJURIES IN RUGBY PLAYERS

### Soft tissue injuries

Soft tissue injuries can usually be treated conservatively by physiotherapists.

### AC joint injuries

AC joint injuries seldom require immediate surgical intervention and can usually be managed conservatively by the physiotherapist and the attending physician. These injuries often respond to conservative management, allowing the player to return to the sport as soon as the pain settles. In more severe injuries like grade IV and V subluxations, surgery is usually advised. The commonly encountered, painful, 'degenerative' AC joint probably results from a grade I subluxation and a concomitant meniscal tear and/or the heavy weightlifting that these players do as part of their training. Treatment is through direct arthroscopy of the AC joint, with debridement of meniscal tears with or without bony decompression.

### Rotator cuff injuries

The majority of rotator cuff injuries in rugby players are sprains/tendonitis. These could be managed conservatively by the physiotherapist and medical attendants; cortisone injection may be required on occasion. If a rotator cuff tear (which is rare in these young players) is suspected, referral to a specialist may be indicated to verify the extent of the injury and possible surgical repair. Some players with complete rotator cuff tears have been able to continue to play, but this is the exception and not the rule. Delaying surgery too long in these cases may lead to a less favorable rehabilitation outcome and permanent joint changes.

### Shoulder dislocations

After reduction of the dislocation, referral to a specialist is advisable to exclude significant damage; labral tears and bony lesions of the glenoid may need to be addressed and, not infrequently, surgery may be indicated to prevent further dislocations. In rugby, conservative treatment is often not ideal as there is a tendency for repeated and more frequent dislocations. Surgery provides the best long-term outcome, especially in professional players.

### Brachial plexus injuries

If such an injury is diagnosed by the attending medical officer, referral to a neurologist may be indicated to confirm the extent and prognosis of the injury. Treatment depends upon the extent of the injury.

### Clavicle fracture

These may usually be managed conservatively with immobilization in a shoulder sling. In cases where the displacement seems too severe, or when there is associated nerve and muscle compromise, consultation with a specialist may be indicated to consider the need for open reduction and internal fixation.

### Pectoralis major rupture

This injury may require surgical repair if the patient is to return to a level of function required to play rugby.

## CONCLUSION

An awareness of specific shoulder injuries in rugby players is necessary for prompt recognition and referral for treatment. With appropriate management, most of the players should be able to return to the sport. Medium- to long-term outcomes of surgical treatment of most of these injuries are favorable.

